# Tirofiban Combination Therapy for Acute Ischemic Stroke: A Systematic Review and Meta‐Analysis

**DOI:** 10.1002/brb3.70508

**Published:** 2025-06-10

**Authors:** Abdullah Bin Kamran, Ahmed Bazil Bin Khalil, Ayesha Muhammad, Hira Arshad, Fatima Nazir, Muhammad Mateen Ali, M. Mairaj Umar, Muhammad Farhan, Sudhair Alam, Javed Iqbal

**Affiliations:** ^1^ Rawalpindi Medical University Rawalpindi Pakistan; ^2^ Shaheed Mohtarma Benazir Bhutto Medical College Lyari Pakistan; ^3^ Department of Neurosurgery Shaheed Saif ur Rehman Teaching Hospital Gilgit Pakistan; ^4^ Nursing Department Communicable Diseases Centre Hamad Medical Corporation Doha Qatar

**Keywords:** acute ischemic stroke, antiplatelet therapy, dual antiplatelet therapy, neurology,stroke, tirofiban

## Abstract

**Introduction:**

Acute ischemic stroke (AIS) is a leading cause of morbidity and mortality globally. Standard antiplatelet therapies, while partially effective, do not fully inhibit all pathways of platelet aggregation, leaving patients at risk of recurrent thrombotic events. Tirofiban, a glycoprotein IIb/IIIa receptor inhibitor, has shown promise as an adjunctive treatment in AIS.

**Methods:**

A comprehensive search was conducted in PubMed, ClinicalTrials.gov, and Cochrane library from inception to July 2024, following PRISMA guidelines. Inclusion criteria comprised randomized controlled trials (RCTs) and comparative observational studies where tirofiban was used as an adjunct to standard antiplatelet therapy. Primary outcomes included symptomatic intracranial hemorrhage (sICH) and favorable modified Rankin scale (mRS) scores at 90 days. Secondary outcomes included National Institute of Health Stroke Scale (NIHSS) scores and all‐cause mortality. Data was analyzed using Review Manager v5.4.1, with random‐effects models employed for all outcomes.

**Results:**

Fifteen studies, comprising 4,457 patients, were included. Tirofiban significantly improved the likelihood of achieving favorable mRS scores (OR 1.65, 95% CI [1.29, 2.11], p = 0.0001), with moderate heterogeneity (I^2^ = 57%, p = 0.006). Tirofiban also significantly reduced NIHSS scores (MD ‐2.08, 95% CI [‐2.77, ‐1.39], p < 0.00001). There was no significant difference in the incidence of sICH between the tirofiban and control groups.

**Conclusion:**

Tirofiban as an adjunct to standard antiplatelet therapy in AIS patients significantly improves functional outcomes and reduces neurological impairment without increasing the risk of sICH.

## Graphical abstract

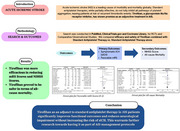

## 1 Introduction

Acute ischemic stroke (AIS) is a critical medical emergency, accounting for approximately 87% of all stroke cases (Malaeb et al. 2023). As the second leading cause of death worldwide and a significant contributor to adult disability, effective management and prevention of AIS are vital. Globally, 15 million individuals experience a stroke each year, with 5 million fatalities and an additional 5 million suffering permanent disability (WHO EMRO | Stroke CaHt). These statistics highlight the pressing need for advancements in stroke treatment strategies to mitigate the burden on patients, families, and healthcare systems.

Current treatment paradigms for AIS primarily focus on the hyper‐acute (0–24 h), acute (1–7 days), and recovery (> 7 days) phases (Li et al. 2022). The efficacy and safety of various therapeutic approaches across these stages are still not entirely understood. Identifying the optimal use of existing and emerging treatments remains a critical challenge in stroke therapy. The cornerstone of acute treatment includes arterial thrombectomy and intravenous thrombolysis (IVT), administered within 4.5 h of symptom onset (Wang et al. 2021). However, for patients who miss this narrow therapeutic window, options are limited, and traditional treatments like low molecular weight heparin are often employed. This highlights a crucial gap in effective interventions for patients who do not meet the criteria for thrombolysis.

Moreover, secondary prevention of AIS remains a crucial aspect of long‐term patient management. Recurrent strokes are a major concern, particularly within the first few months following an initial event (Faure et al. 2020). Standard antiplatelet therapies, while effective to some extent, do not fully inhibit all pathways involved in platelet aggregation (Shah et al. 2022), leaving patients at risk for further thrombotic episodes. Exploring alternative or adjunctive treatments could offer new avenues for enhancing secondary prevention strategies and improving patient outcomes. Recent studies have increasingly focused on tirofiban, a novel antiplatelet agent, as a potential treatment for acute progressive cerebral infarction (Han et al. 2021). Tirofiban is a glycoprotein IIb/IIIa receptor inhibitor that reduces fibrinogen‐platelet bridging, thus potentially preventing arterial thrombosis by inhibiting platelet aggregation (Li et al. 2022). Its mechanism of action involves reversible inhibition of fibrin binding receptors, offering a rapid onset of effect with a short half‐life. This distinct pharmacological profile positions tirofiban as a promising alternative to conventional antiplatelet therapies (Sang et al. 2023).

In China, national guidelines advocate for recombinant tissue plasminogen activator (tPA) (Jilani and Siddiqui 2024) as a standard treatment for hyperacute ischemic stroke (Tam and Tse 2022) due to its efficacy in alleviating neurological impairments. These guidelines also support the use of various secondary prevention strategies, including antithrombotic agents, neuroprotective therapies, statins, and traditional Chinese medicine. Among these, human urinary kallidinogenase (HUK), a bradykinin B1 and B2 receptor agonist, has shown functional advantages in stroke management. Despite these advancements, the treatment landscape remains incomplete, particularly for cases where thrombolysis is no longer viable (Li et al. 2022).

Antiplatelet therapy remains a cornerstone in the secondary prevention of stroke. Dual antiplatelet therapy (DAPT) with clopidogrel and aspirin, as demonstrated in the CHANCE trial (Wang et al. 2015), has been effective in reducing the early risk of stroke following transient ischemic attacks (TIAs) and mild ischemic strokes. Similarly, the THALES trial (Wang et al. 2021) showed that ticagrelor combined with aspirin offers comparable outcomes in preventing stroke and death. Despite these advances, current antiplatelet therapies often fall short of fully inhibiting all pathways involved in platelet aggregation, leaving a gap in the prevention of recurrent thrombotic events.

This meta‐analysis aims to compare the efficacy of tirofiban as an adjunct to standard antiplatelet therapy in managing AIS. By synthesizing data from recent studies, we seek to evaluate whether tirofiban provides a superior alternative to traditional treatments, potentially offering enhanced outcomes for patients who present beyond the acute thrombolysis window or those at high risk of recurrence. Addressing this question is crucial for refining stroke management protocols and improving patient outcomes.

## 2 Methods

### 2.1 Search Strategy

The authors carried out an extensive search on PubMed, Clinicaltrials.gov, and Cochrane library, for studies published in English, starting from inception to July 2024, to identify all existing literature, without any restrictions of time. PRISMA guidelines (Page et al. 2021) were followed for the systematic review. Medical subject heading (MeSH) terms as well as free‐text terms were utilized in each database. The keywords used included “ischemic stroke”, “acute ischemic stroke”, “tirofiban”, “N‐(butylsulfonyl)‐O‐(4‐(4‐piperidyl)butyl)‐L‐tyrosine”, “L 700462”, “aspirin”, and “2‐(acetyloxy)benzoic acid” among many other, with no restrictions on study design. This was followed by a manual search for relevant literature from selected studies and published reviews.

### 2.2 Study Selection and Inclusion Criteria

Reviewers ABK and AB reviewed the titles, abstracts, and full texts of all the articles, after the search, independently. The inclusion criteria was set to: (1) randomized, controlled, parallel trials, or comparative observational studies; (2) intervention group receiving tirofiban either in combination therapy, and the control group receiving standard dual‐antiplatelet therapy with or without placebo or endovascular thrombectomy (EVT); and (3) available outcomes on NIHSS scores, mRS scores, sICH events or mortality rates. Case reports, editorials, review articles, and studies not published in English were excluded.

Any disagreements between the two authors were resolved through a group consensus and a third adjudicator (HA).

### 2.3 Data Extraction and Quality Assessment

Data pertaining to all the study outcomes along with relevant study characteristics were extracted and entered into Microsoft Excel. The recorded data included study authors, design, sample size, population, population age, gender, treatment and control interventions, baseline NIHSS score, and time‐to‐treatment since stroke onset as well as total follow‐up period for each study.

The analysis was carried out in Review Manager v5.4.1. Cochrane's risk of bias (ROB) Assessment tool in Review Manager v5.4.1 was used for randomized, controlled trials whereas the Newcastle‐Ottawa scale was used for the observational retrospective cohorts (Stang 2010).

### 2.4 Outcomes and Analysis

The primary safety and efficacy outcomes were symptomatic intracranial hemorrhage (sICH) events, defined as imaging evidence of ICH with a NIHSS increase of and favorable modified Rankin scale (mRS) score at 90‐day post‐treatment, defined as an mRS score from 0 to 2. The secondary outcomes included the National Institute of Health Stroke Scale (NIHSS) scores for efficacy and All‐cause mortality for safety.

Odds ratios (ORs) were calculated and pooled, via the Mantel‐Haenszel method, for the overall estimate of Tirofiban efficacy through the number of patients with favorable mRS score (0‐2). Risk ratios were calculated with 95% confidence interval for both sICH events and mortality. Mean differences (MD) were calculated for NIHSS scores from individual studies, to obtain a pooled effect measure. Random effects models were prepared for all outcomes. Heterogeneity across the studies was assessed using the Higgins *I^2^* statistic along with Cochran's *Q* test. Significance testing was two‐sided, and *p* < 0.05 was considered statistically significant.

## 3 Results

### 3.1 Search Results

**FIGURE 1:**
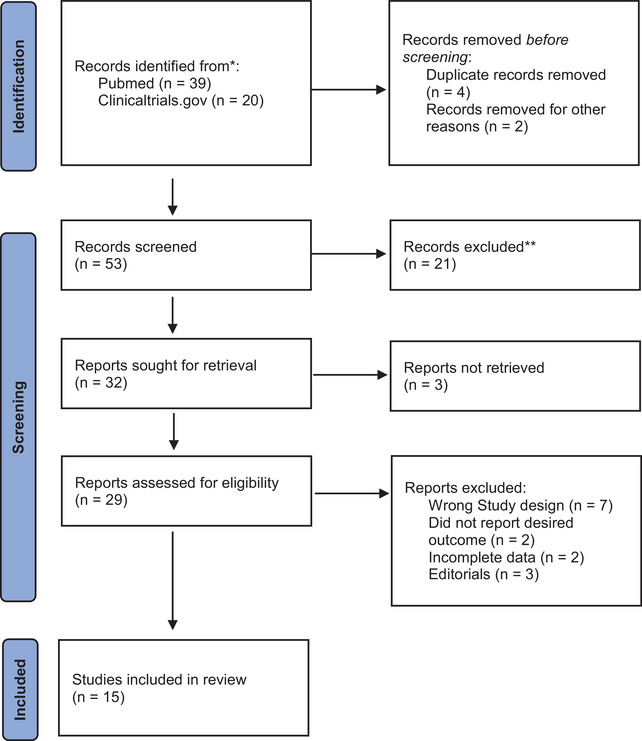
PRISMA flowchart for screening.

Fifty three articles were retrieved from the search after duplicates were discarded, from each database. These articles were then screened by title and abstract, leading to 22 articles that were then reviewed for full‐texts. Seven studies were excluded during the secondary screening. Data from the 15 finalized studies were collected which included 4 Retrospective cohorts and 11 randomized controlled trials (RCTs). The PRISMA flowchart is shown in Figure 1.

### 3.2 Study Characteristics

Fifteen studies were included in the analysis. The patients were divided into two groups. The control group in all studies received standard DAPT with or without a placebo, EVT, or IVT using alteplase in one study (Huo et al. 2021). The treatment group received an initial dose of Tirofiban for 1–3 days followed by standard DAPT consisting of aspirin and/or clopidogrel.

The study characteristics are shown in Table 1. The studies were published between the years 2010 and 2024. There were 11 randomized control trials and 4 cohorts. The general study data extracted from the studies is shown in Table 2.

**TABLE 1:** Study characteristics.

Study author	Study year	Study design	Region	Study duration	Patient population	Treatment intervention	Control intervention	Other intervention(s)^a^
Du	2022	Retrospective cohort	China	May 2018 to December 2019	Progressive ischemic stroke patients	Tirofiban (first at a load of 0.4 µg/(kg·min) for 30 min, and then pumped at 0.1 µg/(kg·min) for 3 days using a micropump)	No tirofiban	Dual anti‐platelet drug therapy^b^
Han	2022	RCT	China	March 2020 to March 2021	Patients aged ≥ 18, with NIHSS score 4–15, within 12 h after stroke onset	Tirofiban (first at a load of 0.4 µg/(kg·min) for 30 mins, and then pumped at 0.1 µg/(kg·min) for 3 d using a micropump) standard treatment (aspirin and/or clopidogrel)	Placebo	Aspirin 100 mg per day for 90 days
Lin	2017	RCT	China	January 2016 to September 2016	AIS patients without arterial occlusion on neurovascular imaging studies	Tirofiban (first at a load of 0.4 µg/(kg·min) for 30 min, and then pumped at 0.1 µg/(kg·min) for 3 d using a micropump) standard treatment (aspirin and/or clopidogrel)	No tirofiban	Dual anti‐platelet drug therapy
Liu	2019	RCT	China	January 2016 to December 2017	AIS patients who underwent IVT with alteplase within 3 h (age 18–85 years) or 3–4.5 h (age 18–80 years) of the onset of stroke; or presence of neurological deficits	Tirofiban 5 µg/kg intravenous bolus, followed by an intravenous infusion of 0.1 µg/kg/min for 24 h	No tirofiban	Dual anti‐platelet drug therapy
Guan	2023	Retrospective cohort	China	January 2020 to December 2021	AIS Patients who underwent EVT	Tirofiban intravenous infusion at a rate of 0.1 µg/kg/min for 12–24 h after a low‐dose intra‐arterial bolus injection (0.25–1 mg)	No tirofiban	EVT followed by dual anti‐platelet drug therapy
Qiu	2020	RCT	China	February 2016 to November 2017	Mild to moderate AIS patients who could not undergo thrombolysis/EVT with platelets ≥ 50×10^9/L	Tirofiban 100 mL, 12500U Heparin, using a micro‐infusion pump at infusion rate of 0.4 µg/kg/min at initial 30 min, followed by infusion at the rate of 0.1 µg/kg/min for 48 h	No tirofiban	Dual anti‐platelet drug therapy
Qiu	2022	RCT	China	October 2018 to October 2021	AIS patients presenting within 24 h of stroke onset, NIHSS score of 30 or less, ASPECTS of 6 or more, and occlusion of the intracranial internal carotid artery or the first or second segment of the middle cerebral artery radiologically confirmed	Tirofiban bolus dose of 10 µg/kg, followed by continuous infusion of 0.15 µg/kg/min for up to 24 h	Placebo	Dual anti‐platelet drug therapy
Siebler	2011	RCT	Germany	2010	AIS patients between 18 and 82 years who were not eligible for thrombolysis and within 3 to 22 h of onset of symptoms with NIHSS scores between 4 and 18	Tirofiban infusion of 0.4 µg/kg body weight/minute over 30 min followed by a continuous infusion of 0.1 µg/kg body weight/minute for 48 h	Placebo	Dual anti‐platelet drug therapy and steroids in some patients
Torgano	2010	RCT	Italy	December 2003 to April 2006	Patients aged 20–90, NIHSS scores of 5–25, symptom duration 1–60 min, without hemorrhage on brain CT, no reported intolerance to ASA or GPIIb/IIIa inhibitors, and onset of stroke < 6h	Tirofiban continuous infusion at 0.6 µg/kg/min for 30 mins followed by 0.15 µg/kg/min for 72 h or less in case of adverse reactions or reduction in the NIHSS score to 0–1.	Aspirin	None
Wu	2019	Retrospective cohort	China	January 2017 to September 2018	AIS patients with age ≥ 18 years with END within the first 24 h after IVT	Tirofiban 5 mg of tirofiban (diluted with 100 mL of normal saline) IV bolus of 0.25 to 0.5 mg (5–10 mL) at a rate of 1 mL/min, followed by a continuous infusion of 0.25 to 0.5 mg/h	No tirofiban	Alteplase 0.9 mg/kg was administered within 4.5 h after symptom onset (IVT)
Zhang	2022	RCT	China	June 2018 to May 2022	AIS patients with END after IVT with rt‐PA	Tirofiban infusion, loading dose of 0.4 µg/kg/min over 30 min followed by a maintenance dose of 0.1 µg/kg/min up to 24 h, starting immediately after END diagnosis	No tirofiban	Dual anti‐platelet drug therapy
Zhang	2023	Retrospective cohort	China	February 2018 to June 2022	AIS patients without IVT within 24 h of onset, diagnosed with a malignant tumor	Tirofiban continuous intravenous administration at a dose of 0.1 µg/kg/min for 48 h before switching to oral aspirin	Aspirin 100 mg QD	None
Zhao	2017	RCT	China	January 2013 to February 2017	AIS patients undergoing EVT	Tirofiban 5 mg (diluted with 100 mL of normal saline) was administrated intra‐arterially at a rate of 1 mL/min. Intravenous tirofiban was continued at a rate of 4 to 5 mL/h for 12 to 24 h, bridged with dual anti‐platelet therapy if no obvious ICH on CT	No tirofiban	EVT followed by dual anti‐platelet drug therapy
Zhao	2024	RCT	China	September 2020 to March 2023	Participants aged 18 to 80 years who presented with AIS within 24 h of symptom onset or time last known well, with a NIHSS score of 4 to 20 points	IV tirofiban was administered at 0.4 µg/kg/min for 30 min, followed by a continuous infusion of 0.1 µg/kg/min for up to 71.5 h	Oral aspirin	None
Zi	2023	RCT	China	October 2020 to June 2022	AIS patients	IV tirofiban 0.4 µg/kg/min for 30 min followed by a continuous infusion of 0.1 µg/kg/min for up 16–48h	Oral aspirin 100 mg per day for 2 days	None

^a^ other intervention(s): interventions common to both groups.

^b^ Dual anti‐platelet drug therapy: Aspirin and/or clopidogrel.

Abbreviations: AIS, Acute Ischemic Stroke; ASPECTS, Alberta Score Program Early CT Score; END, Early Neurological Deterioration; EVT, Endovascular Thrombectomy; IVT, Intravenous Thrombolysis; NIHSS, National Institute of Health Stroke Scale; rt‐PA, Recombinant Tissue Plasminogen Activator.

**TABLE 2:** General study data extracted. Data are either mean ± SD or median (range).

Study author	Study year	Sample size		Population age		Gender		Baseline NIHSS		Time‐to‐treatment		Follow‐up time
		Tirofiban	Control	Tirofiban	Control	Male	Female	Tirofiban	Control	Tirofiban	Control	
Du	2022	75	75	61.88 ± 9.39	60.21 ± 9.73	88	62	6.57 ± 4.27	7.19 ± 4.43	12.43 ± 3.88 h	11.78 ± 3.93 h	90 days
Han	2022	177	180	67 (59‐74)	67 (59‐75)	241	116	5 (4‐8)	6 (4‐8)	4.5 h	4.5 h	90 days
Lin	2017	25	25	63 (47‐82)	70 (44‐89)	34	16	6 (4‐15)	7 (4‐13)	7 (4.5‐23) h	6 (4.5‐24) h	90 days
Liu	2019	60	63	68.05 ± 8.25	67.71 ± 6.72	68	55	9.25 ± 4.52	10.38 ± 4.68	2.75 ± 0.84 h	2.85 ± 0.87 h	90 days
Guan	2023	102	102	69.5 (57‐76)	72.5 (65.8‐80.0)	129	75	13 (10‐17)	14 (10‐22.25)	214 (150‐300) mins	210 (150‐271.25) mins	90 days
Qiu	2020	54	44	68.04 ± 11.47	70.45 ± 10.78	54	31	5.00 ± 1.69	5.23 ± 1.61	< 48 hrs		90 days
Qiu	2022	463	485	68 (58‐74)	67 (57‐75)	557	391	16 (12‐19)	16 (12‐20)	121 (93‐162) min	116 (90‐155) min	90 days
Siebler	2011	131	129	67.6 (34 –81)	65.8 (30 –82)	155	105	6.0 (4–18)	6.0 (4–18)	9.25 h	10.7 h	5 months
Torgano	2010	75	75	71.8 ± 13.7	73.8 ± 8.9	73	77	9(6‐16)	9(7‐14)	4.4 ± 1.06 h	4.4 ± 1.13 h	90 days
Wu	2019	121	66	63.8±13.2	61.5±12.6	126	61	6 (4–11)	7 (4–10)	165.1±34.6 min	157.7±38.7 min	3 months
Zhang	2022	59	14	69.24 ± 14.88	68.21 ± 12.86	39	34	8.90 ± 2.75	8.14 ± 3.51	196.10 ± 53.89 min	209.00 ± 55.17 min	90 days
Zhang	2023	34	41	67.91 ± 10.10	65.24 ± 7.92	56	19	6.85 ± 2.57	7.95 ± 2.99	12.35 ± 5.81 h	12.76 ± 4.53 h	90 days
Zhao	2017	90	90	61.8±13.1	61.8±13.1	129	51	21 (14–32)	19 (15–26)	388 (321–421) min	372 (311–436) min	3 months
Zhao	2024	213	212	64 (56‐70)	64 (56‐71)	301	124	5 (4‐7)	5 (4‐8)	12.5 (7.8‐19.2) h	10.5 (6.6‐21) h	90 days
Zi	2023	606	571	68.0 (58.0–75.0)	68.0 (59.0–76.0)	752	425	9.0 (7.0–10.0)	9.0 (7.0–10.0)	11.3 (7.5–16.5) h	11.5 (7.8–17.1) h	90 days

**FIGURE 2:**
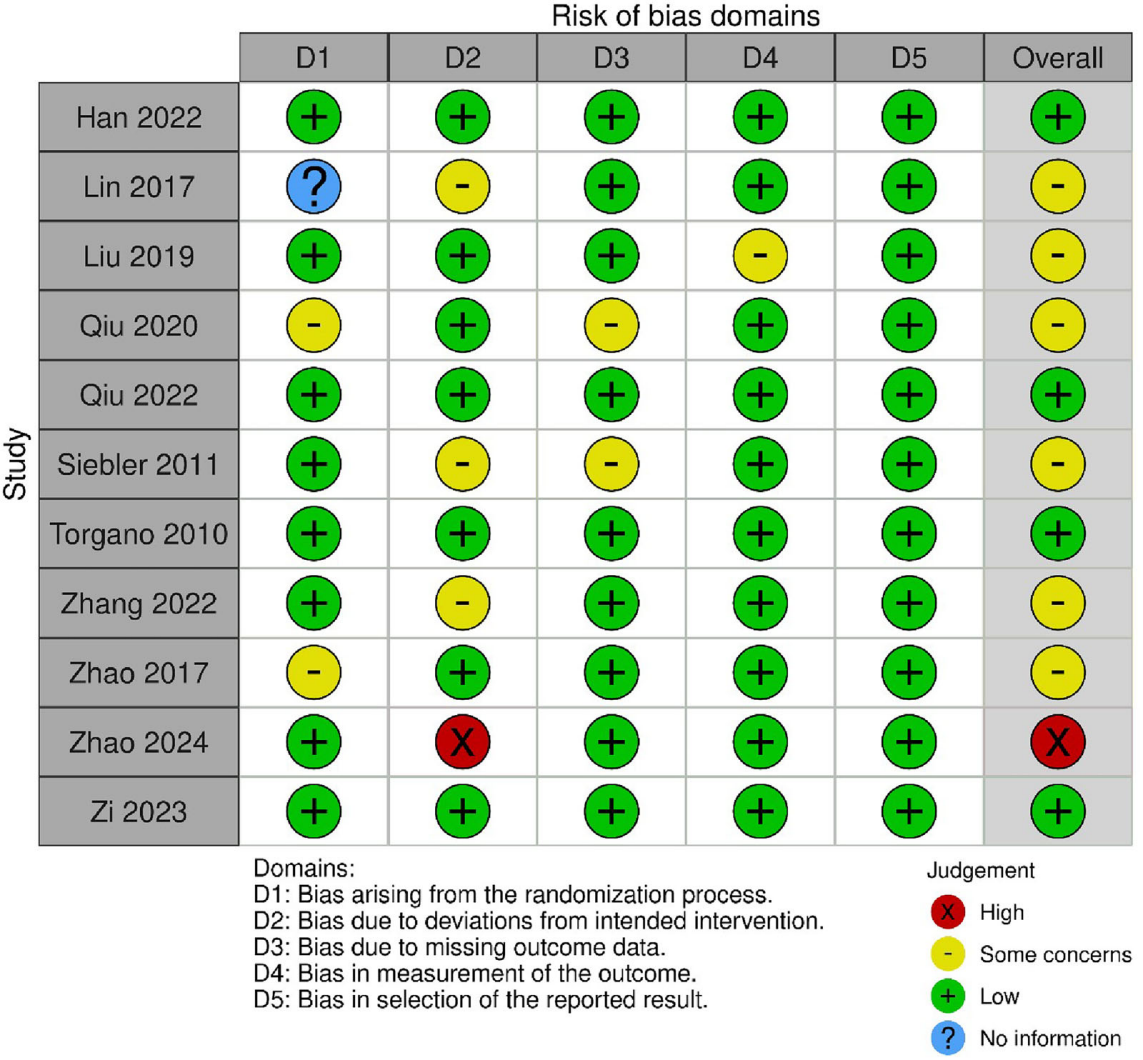
ROB assessment for RCTs.

The number of AIS patients tallied up to a total sample size of 4457. The majority of the studies had a follow‐up time of 90 days. The tirofiban group and the control group were almost equally matched in all domains. Quality assessment of the included retrospective cohort studies is shown in Table 3 using the Newcastle‐Ottowa scale, while the RCTs were assessed using the cochrane ROB as shown in Figure 2. Table 4

**TABLE 3:** Risk of bias using the newcastle‐ottowa scale.

Author ame	Type of study	Selection	Comparability	Exposure/outcome	Overall
Du (202n2)	Retrospective study	****	*	***	8
Guan (2023)	Retrospective study	****	**	***	9
Wu (2019)	Retrospective study	***	*	***	7
Zhang (2023)	Retrospective study	***	**	***	8

**TABLE 4:** A leave‐one‐out analysis for heterogeneity.

Studies excluded	Estimate (OR)	Lower bound	Upper bound	*I^2^*
None	1.649	1.291	2.105	57%
Han	1.646	1.261	2.150	59%
Lin	1.586	1.250	2.011	55%
Liu	1.578	1.238	2.012	55%
Guan	1.607	1.243	2.077	57%
Qiu	1.552	1.235	1.951	51%
Qiu (2)	1.770	1.335	2.348	56%
Torgano	1.739	1.352	2.236	56%
Wu	1.622	1.253	2.101	58%
Zhang	1.580	1.266	1.973	49%
Zhang (2)	1.685	1.303	2.180	60%
Zhao	1.686	1.292	2.199	60%
Zhao (2)	1.697	1.300	2.216	60%
Zi	1.768	1.318	2.372	58%

### 3.3 Efficacy in Obtaining Favorable mRS Scores (0‐2)

**FIGURE 3:**
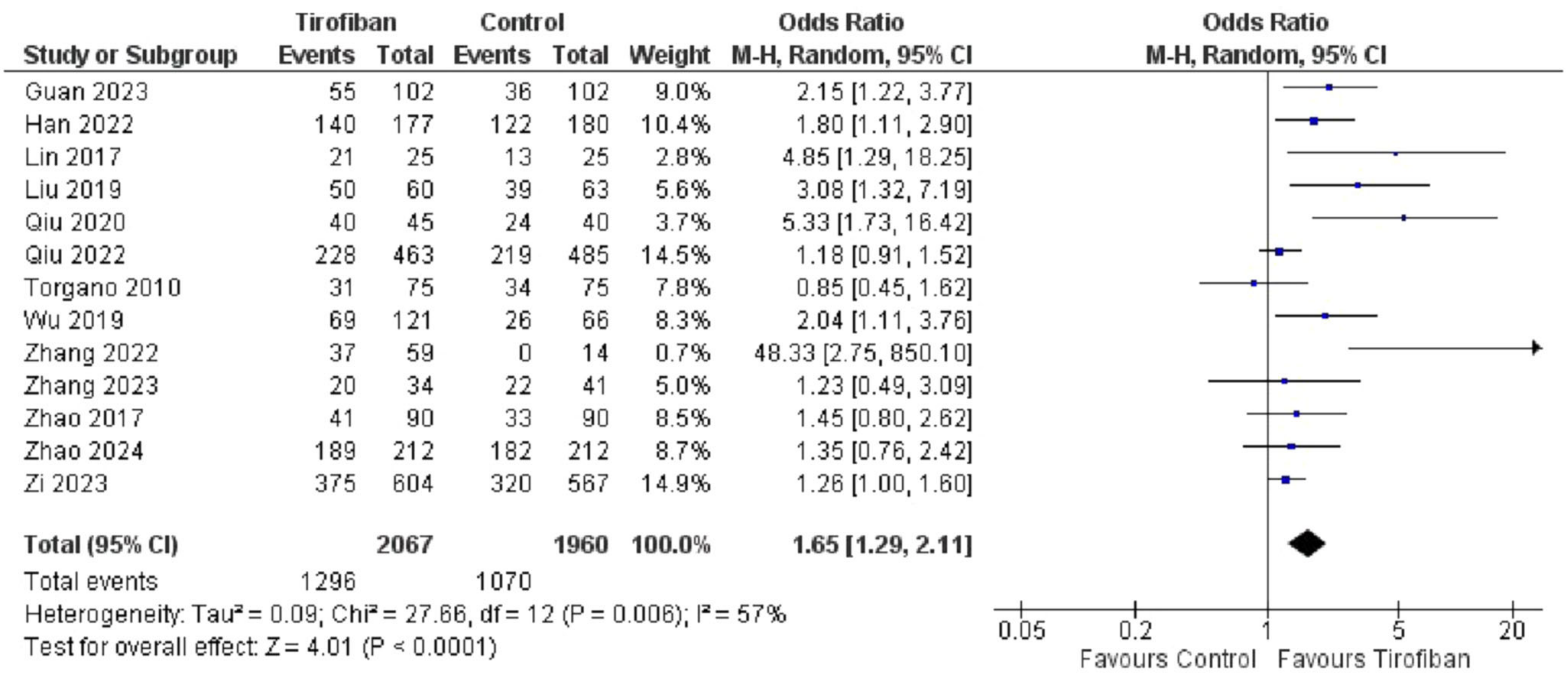
Forest plot for efficacy of Tirofiban in obtaining favorable mRS scores.

The primary outcome for efficacy was number of patients with a favorable mRS score, which was defined as an mRS score of 0 to 2 after treatment. Thirteen studies out of the total 15 mentioned this outcome. 1296 (62.7%) patients out of 2067 in the tirofiban group showed favorable mRS scores as compared to 1070 (54.6%) patients out of 1960 in the control group. The pooled effect showed tirofiban to be effective in reducing mRS scores to 0–2 (*OR 1.65, 95% CI [1.29, 2.11]*) as compared to the control group receiving aspirin and/or clopidogrel alone. The overall effect was significant (*Z = 4.01, P = 0.0001*). However, there was moderate heterogeneity in the studies (*I^2^ = 57%, P = 0.006*). Figure 3 shows the forest plot for this outcome.

### 3.4 Efficacy in Reducing NIHSS Scores

**FIGURE 4:**
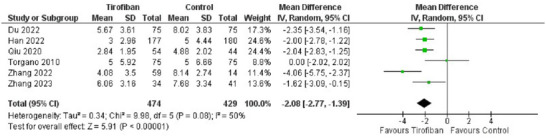
Forest plot for efficacy of Tirofiban in reducing NIHSS scores.

Reduction in NIHSS scores was the secondary outcome for efficacy of tirofiban. A random‐effects model was prepared to evaluate the pooled mean difference (MD) in the tirofiban and control groups. Six studies mentioned NIHSS scores post‐treatment, as part of their results. Where NIHSS scores were reported as medians (IQR), the IQR was converted to SD by dividing by 1.35, considering normal distribution of the data. The pooled effect showed Tirofiban to be extremely effective at reducing NIHSS scores (*MD ‐2.08, 95% CI [‐2.77, ‐1.39]*). The pooled effect was also significant (*Z = 5.91, P < 0.00001*). The forest plot for NIHSS scores is shown in Figure 4.

### 3.5 Symptomatic Intracranial Hemorrhages

**FIGURE 5:**
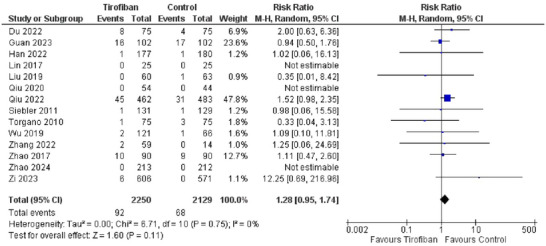
Forest plot for safety of Tirofiban in risk of developing sICH.

The primary safety outcome was the number of patients developing symptomatic ICH, which was defined as evidence of ICH on imaging with an increase in NIHSS score by ≥ 4 points. Fourteen studies reported sICH as an outcome for assessing safety. Three of these studies reported 0 sICHs in both the tirofiban and control groups. Nevertheless, the overall incidence of sICH was higher in the tirofiban group as compared to controls (*4.08%* and *3.19%* for tirofiban and control groups, respectively). This was shown by the pooled effect for the risk of developing sICH (*RR 1.28, 95% CI [0.95, 1.74]*), which favored the control group in terms of safety (Figure 5). However, the overall effect was small and insignificant (*Z = 1.60, P = 0.11*). There was no heterogeneity in the included studies (*I^2^ = 0%*).

### 3.6 All‐Cause Mortality After Treatment

**FIGURE 6:**
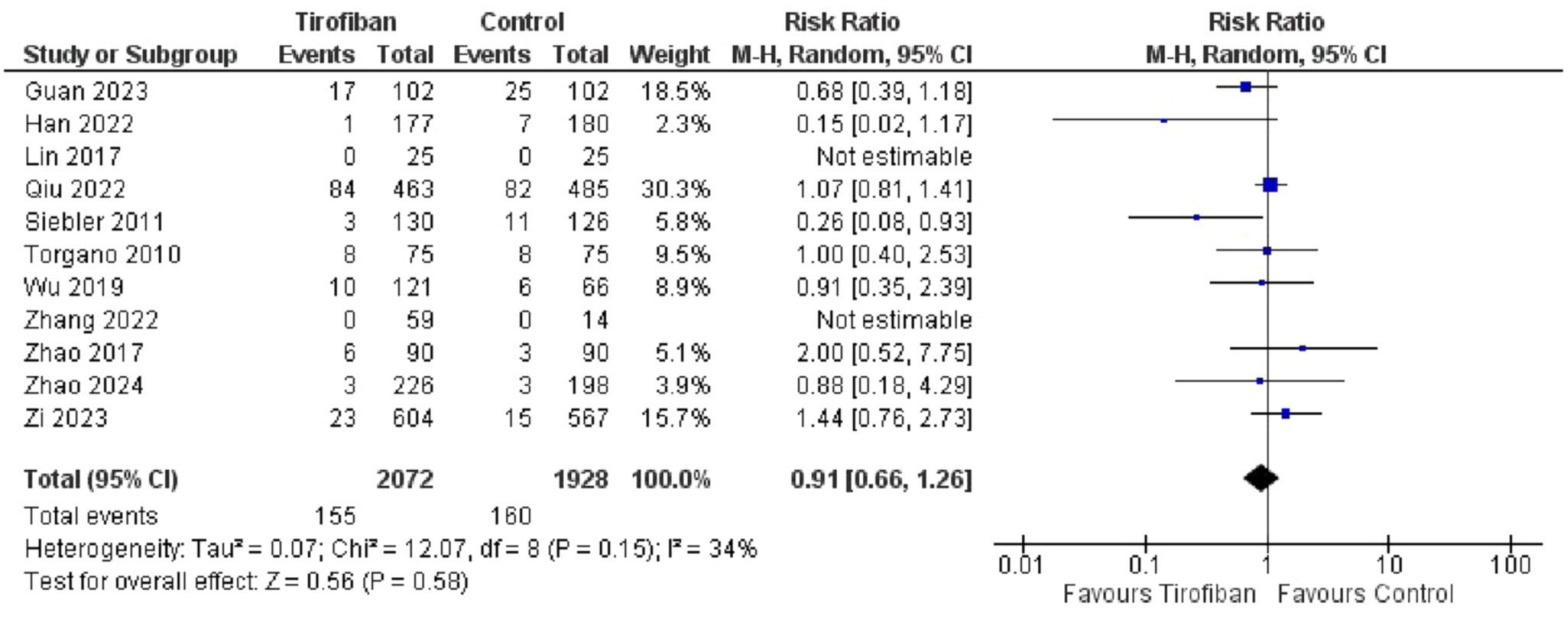
Forest plot for safety of Tirofiban in terms of mortality.

Safety of tirofiban was also evaluated through mortality after treatment. Eleven studies mentioned Mortality as an outcome for safety. Two of these studies mentioned no mortality in either group. Tirofiban group had a lower mortality rate than the Control group (*7.48%* and *8.29%*, respectively). The pooled effect also showed tirofiban to be safer in terms of mortality (*RR 0.91, 95% CI [0.66, 1.26]*). The overall effect was insignificant (*Z = 1.60, P = 0.11*). The summary of these results are shown in Figure 6.

### 3.7 Sensitivity Analysis

**FIGURE 7:**
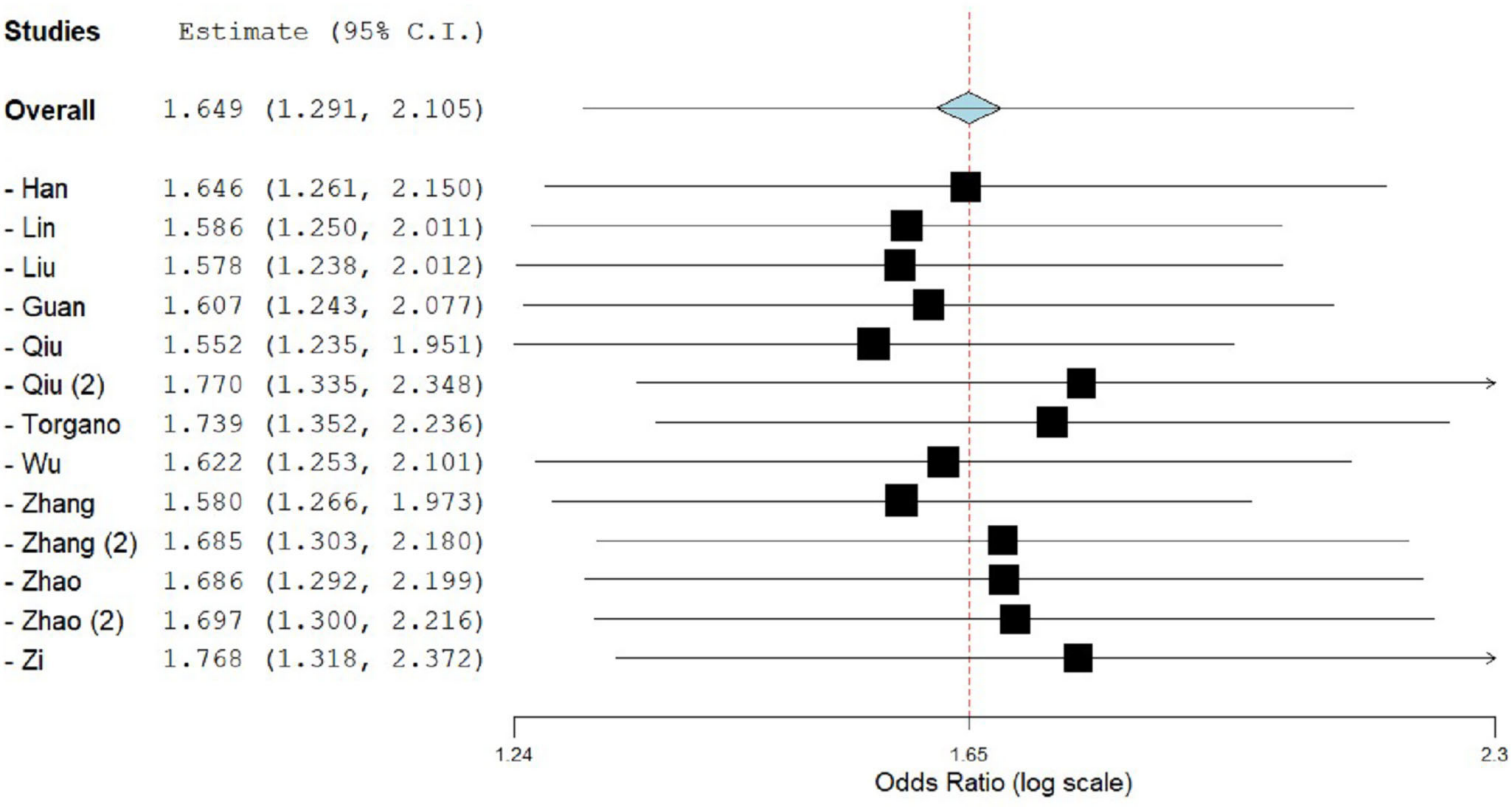
Forest plot for the effect size variation in sensitivity analysis.

Significant heterogeneity was only observed for the favorable mRS scores outcome (*I^2^ = 57%, P = 0.006*). A leave‐one‐out analysis was performed to evaluate the cause of this heterogeneity as shown in Table 4. Removing the study by (Zhang et al. 2022) decreased the heterogeneity the most, bringing it down to (49%). The effect size still shows tirofiban to be efficacious in producing favorable mRS scores (*OR 1.58, 95% CI [1.27, 1.98]*), without the inclusion of the study by *Zhang* et al. The forest plot for variations in the Effect estimate is shown in Figure 7.

## 4 Discussion

The findings of this study provide a comprehensive overview of the efficacy and safety of tirofiban in the management of AIS (Huo et al. 2021). Our analysis included data from 15 studies, comprising 11 RCTs and 4 retrospective cohorts, with a total of 4457 patients. The results demonstrate that tirofiban, when used as an adjunct to standard DAPT (Asdaghi and Romano 2015), significantly improves functional outcomes, as measured by the mRS scores (Banks and Marotta 2007) and NIHSS scores (Kwah and Diong 2014), without a substantial increase in the risk of sICH or all‐cause mortality.

The primary outcome for efficacy was the achievement of favorable mRS scores at 90 days post‐treatment. Our analysis revealed that 62.7% of patients in the tirofiban group achieved favorable mRS scores compared to 54.6% in the control group. This result was statistically significant and indicated that patients treated with tirofiban were 65% more likely to achieve better functional outcomes than those receiving standard DAPT alone. Studies such as Al‐Salihi et al. (2023) show similar results in terms of favorable mRS and NIHHS scores.

The moderate heterogeneity (I2 = 57%) observed suggests some variability in the study populations and methodologies, which is not uncommon in meta‐analyses involving clinical interventions and can be attributed to several factors. Firstly, there is considerable variability in patient populations, such as differences in the severity of ischemic stroke (e.g., varying NIHSS scores), comorbid conditions, and age groups. Some studies focused on patients with mild to moderate AIS, while others involved those with more severe cases or comorbidities like malignancies. Additionally, the timing of treatment initiation varied, with some studies administering tirofiban within hours of symptom onset, while others did so later, potentially influencing the outcomes. Second, the treatment protocols differed in terms of drug dosages, infusion durations, and adjunctive therapies. For instance, some studies used higher bolus doses of tirofiban or extended the duration of infusion, which may have led to different clinical effects.

The secondary efficacy outcome, the reduction in NIHSS scores, further supports the benefit of tirofiban in AIS management. The pooled MD for NIHSS scores indicated a significant improvement in the tirofiban group compared to controls with a highly significant overall effect. This finding emphasizes the role of tirofiban in enhancing neurological recovery post‐stroke, which is crucial for reducing long‐term disability and improving the quality of life for stroke survivors (Grefkes and Fink 2020). The pharmacological action of tirofiban as a glycoprotein IIb/IIIa inhibitor plays a crucial role in preventing platelet aggregation (Al‐Salihi et al. 2023), which is a key factor in the pathophysiology of ischemic stroke. By inhibiting platelet aggregation, tirofiban can reduce the formation of thrombi, thus improving blood flow and reducing the extent of ischemic damage. (Han et al. 2022) This mechanism likely contributes to the observed improvements in mRS and NIHSS scores, supporting its use as an adjunct therapy in AIS. (Yang et al. 2019)

Regarding safety, the incidence of sICH, a critical concern with antithrombotic therapy, was slightly higher in the tirofiban group (4.08%) compared to the control group (3.19%). However, the result was not statistically significant, suggesting that while there is a trend towards increased sICH risk with tirofiban, and it is not substantial. This balance between efficacy and safety is essential in stroke management, where the benefits of improved functional outcomes must be weighed against the risks of adverse events. Given the slightly higher incidence of sICH in the tirofiban group, it is necessary to evaluate the clinical implication of these results under a clinical framework, regardless of statistical insignificance. Careful patient selection, monitoring, and management are warranted to ensure patient safety, above all. All‐cause mortality, another critical safety outcome, was lower in the tirofiban group (7.48%) compared to the control group (8.29%). The pooled risk ratio of this outcome also did not reach statistical significance, indicating any significant difference in mortality risk between the two groups. This suggests that tirofiban does not increase the risk of death and may offer a slight protective effect (Pan et al. 2019), though this result should be interpreted with caution due to the lack of statistical significance.

The observed moderate heterogeneity for the favorable mRS scores outcome prompted a sensitivity analysis, which identified the study by Zhang et al. (2022) as a significant contributor to the variability. Excluding this study reduced heterogeneity to 49%, indicating that differences in study design, patient populations, and treatment protocols can impact the overall results. Clearly, more standardized protocols and larger, multicenter RCTs are needed to confirm these findings and refine treatment guidelines. The inclusion of various study designs, including both RCTs and retrospective cohorts, adds robustness to our analysis but also introduces potential biases.

Retrospective studies, while valuable for providing real‐world evidence, can be prone to selection bias and confounding factors. However, the use of established quality assessment tools, such as the Newcastle‐Ottawa scale for cohort studies and the cochrane ROB tool for RCTs, helped ensure the reliability of our results. Our analysis primarily included studies conducted in China, which may limit the generalizability of the findings to other populations, since stroke care practices, healthcare infrastructure, and patient characteristics can vary significantly across different regions, potentially influencing the outcomes of interventions like tirofiban. The lack of uniformity in the control group intervention and difference in study designs also introduces potential bias into the pooled results, and while appropriate tools have been employed to limit this bias as previously mentioned, there is no way to fully ensure these confounders don't unfairly influence the results of this study. Future research should aim to include more diverse populations and a more standardized form of control group interventions to enhance the external validity of the results.

This study provides strong evidence supporting the efficacy of tirofiban in improving functional outcomes in AIS patients when used alongside standard DAPT. While there is a slight increase in the risk of sICH, the overall safety profile is acceptable, with no significant increase in all‐cause mortality. These findings suggest that tirofiban could be a valuable addition to the current treatment strategies for AIS, particularly in settings where timely and effective intervention is critical. However, further research, particularly in diverse populations and through large‐scale, standardized RCTs, is necessary to confirm these results and optimize treatment protocols.

## 5 Conclusion

In conclusion, this study shows that tirofiban significantly improves functional outcomes in acute ischemic stroke patients when used with standard dual antiplatelet therapy, without significantly increasing the risk of sICH or all‐cause mortality. These findings suggest tirofiban could be a valuable addition to AIS treatment strategies.

## Author Contributions

**Abdullah Bin Kamran**: conceptualization, data curation, writing–original draft, formal analysis. **Ahmed Bazil Bin Khalil**: data curation, methodology, formal analysis. **Ayesha Muhammad**: methodology, writing–original draft, writing–review and editing. **Hira Arshad**: methodology, writing–review and editing, writing–original draft. **Fatima Nazir**: methodology, writing–original draft, writing–review and editing. **Muhammad Mateen Ali**: writing–original draft, writing–review and editing. **M. Mairaj Umar**: writing–review and editing, writing–original draft. **Muhammad Farhan**: writing–original draft, writing–review and editing. **Sudhair Alam**: writing–review and editing, supervision. **Javed Iqbal**: writing–review and editing, supervision.

## Conflicts of Interest

The authors declare no conflicts of interest.

## Peer Review

The peer review history for this article is available at https://publons.com/publon/10.1002/brb3.70508.

## Data Availability

The data that support the findings of this study are available from the corresponding author upon reasonable request.
